# Stress hyperglycemia is associated with in‐hospital mortality in patients with diabetes and acute ischemic stroke

**DOI:** 10.1111/cns.13764

**Published:** 2022-01-27

**Authors:** Donghua Mi, Zixiao Li, Hongqiu Gu, Yingyu Jiang, Xingquan Zhao, Yilong Wang, Yongjun Wang

**Affiliations:** ^1^ Department of Vascular Neurology Beijing Tiantan Hospital, Capital, Medical University Beijing China; ^2^ China National Clinical Research Center for Neurological Diseases Beijing China; ^3^ Tiantan Clinical Trial and Research Center for Stroke Department of Neurology Beijing Tiantan Hospital Capital Medical University Beijing China; ^4^ Center for Stroke Beijing Institute for Brain Disorders Beijing China; ^5^ Beijing Key Laboratory of Translational Medicine for Cerebrovascular Disease Beijing China

**Keywords:** diabetes, hyperglycemia, ischemia, mortality, physiological, stress, stroke

## Abstract

**Background and Objective:**

Stress hyperglycemia may occur in diabetic patients with acute severe cerebrovascular disease, but the results regarding its association with stroke outcomes are conflicting. This study aimed to examine the association between stress‐induced hyperglycemia and the occurrence of in‐hospital death in patients with diabetes and acute ischemic stroke.

**Research Design and Methods:**

All data were from the Chinese Stroke Center Alliance (CSCA) database and were collected between 2016 and 2018 from >300 centers across China. Patients’ demographics, clinical presentation, and laboratory data were extracted from the database. The primary endpoint was in‐hospital death. The ratio of fasting blood glucose (FBG) to HbA1c was calculated, that is, the stress‐induced hyperglycemia ratio (SHR), to determine stress hyperglycemia following acute ischemic stroke.

**Results:**

A total of 168,381 patients were included. The mean age was 66.2 ± 10.7, and 77,688 (43.0%) patients were female. The patients were divided into two groups: survivors (n = 167,499) and non‐survivors (n = 882), as well as into four groups according to their SHR quartiles (n = 42,090–42,099/quartile). There were 109 (0.26%), 142 (0.34%), 196 (0.47%), and 435 (1.03%) patients who died in the Q1, Q2, Q3, and Q4 quartiles, respectively. Compared with Q1 patients, the death risk was higher in Q4 patients (odds ratio (OR) = 4.02) (adjusted OR = 1.80, 95% confidence interval [CI] = 1.10–2.92, p = 0.018 after adjustment for traditional cardiovascular risk factors). The ROC analyses showed that SHR (AUC = 0.667, 95% CI: 0.647–0.686) had a better predictive value for mortality than that of fasting blood glucose (AUC = 0.633, 95% CI: 0.613–0.652) and HbA1c (AUC = 0.523, 95% CI: 0.504–0.543).

**Conclusions:**

The SHR may serve as an accessory parameter for the prognosis of patients with diabetes after acute ischemic stroke. Hyperglycemia in stroke patients with diabetes mellitus is associated with a higher risk of in‐hospital death.

## Graphical abstract

Stress‐induced hyperglycemia ratio may serve as an accessory parameter for the prognosis of patients with diabetes after acute ischemic stroke. Hyperglycemia in stroke patients with diabetes mellitus is associated with a higher risk of in‐hospital death.

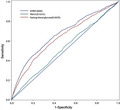

## 1 BACKGROUND

Acute stress hyperglycemia is a common manifestation found in patients presenting to the emergency room with acute cerebrovascular disease. Acute stress hyperglycemia is not only associated with the severity of stroke^1^, ^2^ but also with poor outcomes of stroke, especially in patients without diabetes mellitus.^3^ On the other hand, the association between acute hyperglycemia and the outcomes of patients with diabetes mellitus is controversial, not only for stroke but also for other critical illnesses.^1^, ^4^, ^5^ Indeed, a meta‐analysis of stroke and hyperglycemia demonstrated that stress hyperglycemia in non‐diabetic patients was associated with an increased risk of mortality after stroke (pooled relative risk (RR) = 3.07, 95% confidence index (CI): 2.50–3.79), but this was not observed in patients with a history of diabetes.^6^ This phenomenon is supported by other cohort studies^4^, ^7^ and was also observed in other critical illnesses, that is, that acute hyperglycemia with pre‐existing diabetes mellitus led to lower mortality and shorter length of ICU stay than in patients without diabetes.^6^, ^8^, ^9^

There is no unified definition of stress hyperglycemia,^10^ and the patients are generally classified as known diabetes, newly diagnosed diabetes, and hospital‐related hyperglycemia.^3^, ^11^, ^12^ Most of the previous studies simply used fasting glucose or initial blood glucose at admission to determine the presence of stress hyperglycemia, without considering the usual glucose levels before stroke onset. This might explain why stress hyperglycemia cannot predict the outcome of stroke in patients with a history of diabetes because of high background glucose levels. Therefore, background blood glucose levels should be considered when assessing the relationship between stress hyperglycemia and the outcomes of critical illness, especially for patients with pre‐existing diabetes. The stress hyperglycemia ratio (SHR) is a new method for determining blood glucose stress. It also considers glycated hemoglobin (HbA1c) (which represents the blood glucose levels over the last 2–3 months) and random blood glucose after the stress events.^13^

A study on acute ischemic stroke in patients with diabetes showed that the use of the glycemic gap and SHR as indicators of stress hyperglycemia could be better predictors for the severity and poor outcome of stroke,^14^ but this study was a single‐center, small sample study, limiting the generalizability of its results. In addition, most previous studies used admission glucose, which can be influenced by the diabetic status and the food consumed over the previous hours.^10^, ^13^, ^15^, ^16^, ^17^ Therefore, fasting blood glucose (FBG) instead of random or admission glucose could be a more reliable marker, as previously suggested.^18^ China is a large country with a high burden of cerebrovascular diseases.^19^, ^20^ With the support of the Chinese government, an important database was built at Tiantan Hospital. The aim of the present study was to examine the association between stress‐induced hyperglycemia and the occurrence of in‐hospital death in patients with diabetes and acute ischemic stroke.

## 2 METHODS

### 2.1 Study participants

All data for the present study were from the Chinese Stroke Center Alliance (CSCA) database and were collected between August 01, 2015, and July 31, 2019, from 1476 centers across China. The National Clinical Research Center for Nervous System Diseases of Beijing Tiantan Hospital is mandated to manage the CSCA. The CSCA is funded by the Chinese government, and the data are not accessible to the general public. The CSCA aims to establish a continual stroke registry of patients with stroke in China. It contains the data of millions of patients with stroke from all over China, and it aims to help reduce the burden of stroke in China.^21^ The present study was approved by the ethics committee of Beijing Tiantan Hospital. Individual consent was waived by the committee because the data were anonymized.

The patient population of the CSCA includes (a) patients ≥18 years of age; (b) primary diagnosis of stroke or transient ischemic attack confirmed by brain computed tomography (CT) or magnetic resonance imaging (MRI); (c) within 7 days of onset; and (d) admitted directly to wards or from the emergency department.

The study population included patients with acute cerebral infarction within 72 h of onset and with a previous history of diabetes or previous use of hypoglycemic drugs for > 6 months. Diabetes diagnosis was based on a self‐reported history of diabetes confirmed by the medical records. Potential patients were excluded from the study if they had incomplete information on in‐hospital mortality or had missing HbA1c and FBG data.

### 2.2 Data collection and outcome assessment

In the CSCA, data are directly entered by each center using a web‐based patient data collection interface by trained registrars.^21^ Baseline information was extracted from the database, including demography, vascular risk factors, infarction location, laboratory data, and clinical data. Vascular risk factors included a medical history of hypertension, atrial fibrillation, coronary artery disease, and current smoking. Clinical data included systolic and diastolic blood pressure, baseline National Institutes of Health Stroke Score (NIHSS), leukocytes, alcohol intake, body mass index (BMI), and brain natriuretic peptide (BNP) at admission. Current smoking was defined as smoked at least one cigarette per day for the previous year or more. Laboratory data included baseline HbA1c, fasting blood sugar, and routine blood biochemical variables that were obtained following an 8–12 h fast within the first 24 h after admission. Laboratory data were from the certified central hospital laboratories.

### 2.3 Assessment of initial fasting glucose levels, HbA1c, and SHR

To determine the presence of stress hyperglycemia following an acute ischemic stroke, the ratio of AG (random glucose at admission) to HbA1c was used, which is named the SHR.^13^ In order to eliminate the confounding effect of food, AG in the formula was replaced by FBG (fasting blood glucose) in the present study, as supported by a previous study.^18^ HbA1c was measured within 24 h after admission using high‐performance liquid chromatography (HPLC) (G8 HPLC Analyzer; Tosoh Bioscience). The analyses were conforming with the Diabetes Control and Complications Trial and National Glycohemoglobin Standardization Program (NGSP) standards.

### 2.4 Grouping

The included patients were separated into four groups based on the quartiles of SHR. The ranges of the Q1, Q2, Q3, and Q4 of SHR were 0–0.90, 0.90–1.08, 1.08–1.32, and > 1.32.

### 2.5 Statistical analysis

The data were tested for normal distribution using the Kolmogorov‐Smirnov test. The continuous variables were expressed as means ± SD or as medians with interquartile ranges according to their distribution (normal or skewed). Statistical comparisons of continuous variables were performed using Student's t test or Wilcoxon rank‐sum test (comparisons of two groups), or ANOVA or the Kruskal‐Wallis U test (comparison of more than two groups). Categorical variables were expressed as numbers and percentages and analyzed using the chi‐square test or Fisher's exact test. We evaluated the association between SHR and in‐hospital death using multivariable logistic regression analysis adjusted for potential confounders, including age, sex, BMI, NIHSS on admission, hypertension, atrial fibrillation, previous ischemic stroke, previous myocardial infarction, SAH, antiplatelets, anticoagulation, lipid‐lowering drug, smoking, alcohol, LDL‐C, FBG, HbA1c, eGFR, HCY, systolic blood pressure, and diastolic blood pressure. Receiver operating characteristics (ROC) curves were generated to examine the predictive value of SHR, FBG, and HbA1c, based on the area under the ROC curve (AUC). All statistical analyses were performed using SAS 9.4 (SAS Institute). A SAS macro named %ggBaseline was used to analyze and report the baseline characteristics automatically.^22^ P‐values < 0.05 (two‐sided) were deemed statistically significant.

## 3 RESULTS

### 3.1 Characteristics of the patients

**FIGURE 1:**
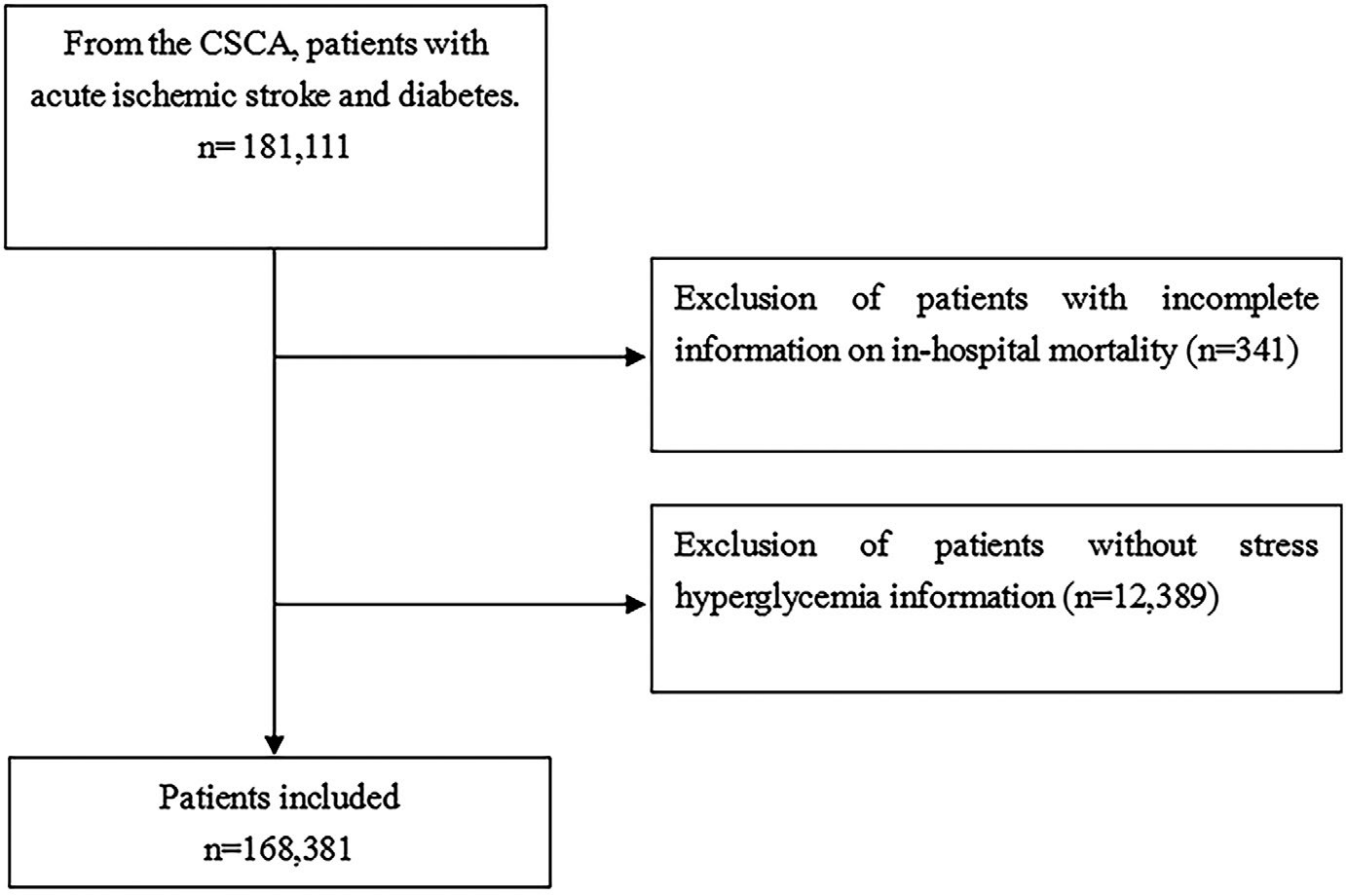
Patient flow diagram. CSCA, China Stroke Center Alliance

A total of 181,111 diabetic patients with acute ischemic stroke were available in the CSCA database; 341 patients were excluded for incomplete data regarding in‐hospital mortality, and 12,389 patients were excluded for missing glucose data. Therefore, 168,381 patients were included in this study (Figure 1). The mean age was 66.2 ± 10.7, and 77,688 (43.0%) patients were female.

### 3.2 Characteristics of the patients according to the vital status

The patients were divided into two groups according to the outcome during hospitalization: survivors (n = 167,499) and non‐survivors (n = 882). The characteristics of the two groups are shown in Table 1. The patients in the non‐survivor group were more likely to display traditional cardiovascular risk factors such as being older (*p *< 0.0001), hypertension (*p* = 0.0004), history of myocardial infarction (*p* < 0.0001), higher initial fasting blood glucose (*p* < 0.0001), higher HbA1c (*p* = 0.018), lower eGFR (*p* < 0.0001), higher homocysteine (*p* < 0.0001), and higher systolic blood pressure (*p* < 0.0001), as well as factors associated with poor stroke prognosis, such as higher NIHSS (*p* < 0.0001), history of transient ischemic attack (*p* = 0.024), history of stroke (*p* < 0.0001), cerebral hemorrhage (*p* = 0.033), subarachnoid hemorrhage (*p* = 0.004). Regarding treatments, the non‐survivors were more likely to be on antiplatelet (*p* = 0.001), anticoagulants (*p* < 0.0001), and lipid‐lowering drugs (*p* = 0.003) and to have received reperfusion therapy (*p* < 0.001), suggesting that they had the indications for such treatments. Otherwise, the non‐survivors showed characteristics usually associated with a better prognosis, such as not smoking (*p* < 0.0001) and not drinking (*p* = 0.007). Regarding the specific objective of the present study, the non‐survivors had a higher SHR compared with the survivor group (*p* < 0.0001).

**TABLE 1:** Clinical features of the survivors and non‐survivors

Variables	All (N = 168,381 [100%])	Survivors (N = 167,499 [99.5%])	Non‐survivors (N = 882 [0.5%])	*p*
Demography				
Age (years)	66.2 ± 10.7	66.1 ± 10.7	73.2 ± 11.1	<0.0001
Female	72,422 (43.0)	72,017 (43.0)	405 (45.9)	0.0803
Body mass index (kg/^2^)	24.5 ± 4.7	24.5 ± 4.7	24.4 ± 6.6	0.6065
Previous history				
Previous transient ischemic attack	2871 (1.7)	2854 (1.7)	17 (1.9)	0.0238
Previous ischemic stroke	65,665 (39.0)	65,230 (38.9)	435 (49.3)	<0.0001
Previous myocardial infarction	4436 (2.6)	4345 (2.6)	91 (10.3)	<0.0001
Cerebral hemorrhage	4066 (2.4)	4035 (2.4)	31 (3.5)	0.0329
Subarachnoid hemorrhage	467 (0.3)	460 (0.3)	7 (0.8)	0.0035
Risk factors				
Glasgow Coma Scale	15.0 (1.0–15.0)	15.0 (1.0–15.0)	5.0 (5.0–5.0)	0.5067
NIH Stroke Score	3.0 (2.0–6.0)	3.0 (2.0–6.0)	15.0 (9.0–21.0)	<0.0001
Hypertension	130,265 (77.4)	129,539 (77.3)	726 (82.3)	0.0004
Atrial fibrillation	7988 (4.7)	7757 (4.6)	231 (26.2)	<0.0001
Smoking	32,647 (19.4)	32,544 (19.4)	103 (11.7)	<0.0001
Alcohol	35,369 (21.0)	35,213 (21.0)	156 (17.7)	0.0073
Previous drugs				
Antiplatelet	49,346 (29.3)	49,047 (29.3)	299 (33.9)	0.0011
Anticoagulation	8424 (5.0)	8358 (5.0)	66 (7.5)	<0.0001
Lipid‐lowering drug	26,620 (15.8)	26,458 (15.8)	162 (18.4)	0.0028
Reperfusion therapy	9191 (5.5)	9003 (5.4)	188 (21.3)	<0.0001
Biochemical indexes				
Low‐density lipoprotein (mmol/L)	2.8 ± 1.3	2.8 ± 1.3	2.9 ± 1.6	0.9406
Fasting blood glucose (mmol/L)	8.1 (6.4–10.9)	8.1 (6.4–10.9)	10.2 (7.3–14.0)	<0.0001
HbA1c (%)	7.6 (6.5–9.2)	7.6 (6.5–9.2)	7.4 (6.3–9.0)	0.0178
eGFR (ml/min/1.73 m^2^)	1.1 (0.9–1.3)	1.1 (0.9–1.3)	1.3 (1.0–1.8)	<0.0001
Homocysteine (µmol/L)	98.7 ± 97.8	98.8 ± 98.0	75.8 ± 41.7	<0.0001
Diastolic blood pressure (mmHg)	13.8 ± 6.8	13.8 ± 6.8	15.2 ± 8.3	<0.0001
HbA1c (%)	151.1 ± 22.5	151.1 ± 22.4	155.3 ± 26.8	<0.0001
Homocysteine (µmol/L)	86.0 ± 13.1	86.0 ± 13.1	86.6 ± 15.5	0.2755
SHR	1.1 (0.9–1.3)	1.1 (0.9–1.3)	1.3 (1.0–1.7)	<0.0001

### 3.3 SHR and in‐hospital death

The patients were divided into four groups according to their SHR quartiles. The characteristics are presented in Table 2. We identified a potential association between the occurrence of in‐hospital death and SHR in diabetic patients with acute ischemic stroke in the unadjusted (P for trend <0.0001) and adjusted (P for trend = 0.0141) models (Table 3). Multicollinearity was investigated, and no multicollinearity was found. There were 109 (0.26%), 142 (0.34%), 196 (0.47%), and 435 (1.03%) patients who died in the Q1, Q2, Q3, and Q4 quartiles, respectively. Q1 was used as the reference. Although the P for trend was significant, there were significant differences in Q2 in the unadjusted analysis (odds ratio [OR] = 1.30, *p* < 0.0001) but not in the adjusted analysis (OR = 1.20, 95% confidence interval [CI] = 0.79–1.81, *p* = 0.382), and there were significant differences for Q3 (adjusted OR = 1.51, 95% confidence interval [CI] = 1.00–2.26, *p* = 0.048). Compared with Q1, the death risk was increased in Q4 (OR = 4.02) (adjusted OR = 1.80, 95% confidence interval [CI] = 1.10–2.92, *p* = 0.018) after adjusting for age, sex, BMI, NIHSS on admission, hypertension, atrial fibrillation, previous ischemic stroke, previous myocardial infarction, SAH, antiplatelet, anticoagulation, lipid‐lowering drug, smoking, alcohol, LDL‐C, FBG, HbA1c, eGFR, HCY, systolic blood pressure, diastolic blood pressure, and reperfusion therapeutic in a Cox regression model (P for trend = 0.014).

**TABLE 2:** Characteristics according to the stress hyperglycemia states measured by the glucose‐to‐HbA1c ratio

Variables	Q1 (N = 42,099 [0–0.90])	Q2 (N = 42,094 [0.90–1.08])	Q3 (N = 42,098 [1.08–1.32])	Q4 (N = 42,090 [>1.32])	*p*
Demography					
Age (years)	67.1 ± 10.6	66.4 ± 10.6	65.7 ± 10.8	65.5 ± 10.8	<0.0001
Female	17,708 (42.1)	17,753 (42.2)	18,152 (43.1)	18,809 (44.7)	<0.0001
Body mass index (kg/m^2^)	24.29 ± 4.57	24.58 ± 4.77	24.58 ± 4.13	24.55 ± 5.23	<0.0001
Previous history					
Previous transient ischemic attack	768 (1.8)	682 (1.6)	692 (1.6)	729 (1.7)	0.1894
Previous ischemic stroke	16,689 (39.6)	16,320 (38.8)	16,060 (38.1)	16,596 (39.4)	0.0003
Previous myocardial infarction	1177 (2.8)	1161 (2.8)	1004 (2.4)	1094 (2.6)	<0.0001
Cerebral hemorrhage	1064 (2.5)	1030 (2.4)	983 (2.3)	989 (2.3)	0.2269
Subarachnoid hemorrhage	146 (0.3)	120 (0.3)	100 (0.2)	101 (0.2)	0.0075
Risk factors					
Glasgow coma scale	11.00 (1.00–15.00)	15.00 (1.00–15.00)	15.00 (12.00–15.00)	15.00 (13.50–15.00)	0.1752
NIH Stroke Scale	3.00 (2.00–6.00)	3.00 (2.00–6.00)	3.00 (2.00–6.00)	4.00 (2.00–7.00)	<0.0001
Hypertension	32,739 (77.8)	3,3046 (78.5)	32,510 (77.2)	31,970 (76.0)	<0.0001
Atrial fibrillation	1983 (4.7)	1993 (4.7)	1877 (4.5)	2135 (5.1)	0.0001
Smoking	8039 (19.1)	8221 (19.5)	8439 (20.0)	7948 (18.9)	<0.0001
Alcohol	8096 (19.2)	8861 (21.1)	9304 (22.1)	9108 (21.6)	<0.0001
Previous drugs					
Antiplatelet	12,941 (30.7)	12,729 (30.2)	12,011 (28.5)	11,665 (27.7)	<0.0001
Anticoagulation	2223 (5.3)	2131 (5.1)	1898 (4.5)	2172 (5.2)	<0.0001
Lipid lowering	6915 (16.4)	6895 (16.4)	6506 (15.5)	6304 (15.0)	<0.0001
Reperfusion therapy	1954 (4.6)	2193 (5.2)	2258 (5.4)	2786 (6.6)	<0.0001
Biochemical indexes					
Low‐density lipoprotein (mmol/L)	2.73 ± 1.28	2.82 ± 1.26	2.87 ± 1.23	2.96 ± 1.39	<0.0001
Fasting blood glucose (mmol/L)	5.86 (4.94–7.08)	7.26 (6.25–8.70)	8.90 (7.60–10.80)	12.55 (10.04–15.50)	<0.0001
HbA1c (%)	7.90 (6.79–9.69)	7.30 (6.30–8.70)	7.50 (6.50–9.10)	7.60 (6.20–9.20)	<0.0001
eGFR (ml/min/1.73 m^2^)	97.84 ± 126.93	99.16 ± 92.42	100.16 ± 83.99	97.74 ± 80.83	<0.0001
Homocysteine (µmol/L)	13.97 ± 6.71	13.97 ± 6.72	13.82 ± 6.72	13.54 ± 6.92	<0.0001
Systolic blood pressure (mmHg)	149.50 ± 21.97	150.42 ± 21.91	151.79 ± 22.27	152.77 ± 23.48	<0.0001
Diastolic blood pressure (mmHg)	84.62 ± 12.84	85.83 ± 12.83	86.60 ± 13.03	87.14 ± 13.69	<0.0001

**TABLE 3:** Adjusted hazard ratios of outcomes at 12 months according to glucose‐to‐HbA1c ratio quartiles

Covariate	Level	Death N (%)	Crude Results			Adjusted Results^a^		
			Odds Ratio (95% CI)	*p*	P for trend^b^	Odds Ratio (95% CI)	*p*	P for trend^b^
Glucose‐to‐HbA1c ratio groups	Q1	109 (0.26)	–	–	<0.0001	–	–	0.0141
	Q2	142 (0.34)	1.304 (1.016–1.674)	<0.0001		1.201 (0.796–1.813)	0.3822	
	Q3	196 (0.47)	1.802 (1.425–2.278)	0.6677		1.505 (1.004–2.256)	0.0477	
	Q4	435 (1.03)	4.023 (3.260–4.965)	<0.0001		1.797 (1.104–2.924)	0.0184	

^a^ Adjusted for age, sex, BMI, NIHSS on admission, hypertension, atrial fibrillation, previous ischemic stroke, previous myocardial infarction, SAH, antiplatelet, anticoagulation, lipid‐lowering drug, smoking, alcohol, LDL‐C, FBG, HbA1c, eGFR, HCY, systolic blood pressure, diastolic blood pressure, and reperfusion therapeutic.

^b^ Test for trend based on the variable containing the median value for each quintile.

Table 4 shows the subgroup analyses according to the type of stroke. After adjusting for age, sex, BMI, NIHSS on admission, hypertension, atrial fibrillation, previous ischemic stroke, previous myocardial infarction, SAH, antiplatelet, anticoagulation, lipid‐lowering drug, smoking, alcohol, LDL‐C, FBG, HbA1c, eGFR, HCY, systolic blood pressure, and diastolic blood pressure in a Cox regression model, compared with Q1, Q4 had a higher risk of death in patients with ischemic stroke (OR = 2.27, 95% CI: 1.18–4.40, *p* = 0.015), and Q3 had a higher risk of death in patients with reperfusion therapy (OR = 2.91, 95% CI: 1.12–7.57, *p* = 0.029).

**TABLE 4:** Subgroup analyses for adjusted hazard ratios of outcomes at 12 months according to glucose‐to‐HbA1c ratio quartiles

Covariate	Level	Death N (%)	Crude results			Adjusted results^a^		
			Odds ratio (95% CI)	*p*‐value	P for trend^b^	Odds ratio (95% CI)	*p*‐value	P for trend^b^
History of ischemic stroke								
Glucose‐to‐HbA1c ratio groups	Q1	57 (0.34)	–	–	<0.0001	–	–	0.0076
	Q2	65 (0.40)	1.167 (0.817–1.666)	0.3961		1.207 (0.668–2.183)	0.5331	
	Q3	92 (0.57)	1.681 (1.207–2.341)	0.0021		1.593 (0.899–2.824)	0.1109	
	Q4	221 (1.33)	3.938 (2.941–5.273)	<0.0001		2.274 (1.176–4.397)	0.0146	
MI								
Glucose‐to‐HbA1c ratio groups	Q1	14 (1.19)	–	–	<0.0001	–	–	0.3871
	Q2	11 (0.95)	0.795 (0.359–1.758)	0.5703		0.510 (0.136–1.919)	0.3196	
	Q3	24 (2.39)	2.034 (1.047–3.954)	0.0362		1.493 (0.437–5.102)	0.5223	
	Q4	42 (3.84)	3.317 (1.801–6.107)	0.0001		1.499 (0.280–8.013)	0.6360	
Hemorrhage								
Glucose‐to‐HbA1c ratio groups	Q1	5 (0.47)	–	–	0.0070	–	–	0.0506
	Q2	4 (0.39)	0.826 (0.221–3.084)	0.7759		3.253 (0.192–55.201)	0.4142	
	Q3	8 (0.81)	1.738 (0.567–5.330)	0.3338		1.014 (0.038–26.991)	0.9935	
	Q4	14 (1.42)	3.042 (1.092–8.477)	0.0333		16.826 (0.771–367.381)	0.0728	
Subarachnoid								
Glucose‐to‐HbA1c ratio groups	Q1	1 (0.68)	–	–	0.0513	–	–	–
	Q2	1 (0.83)	1.218 (0.075–19.688)	0.8893		–	–	
	Q3	1 (1.00)	1.465 (0.091–23.693)	0.7882		–	–	
	Q4	4 (3.96)	5.980 (0.658–54.310)	0.1121		–	–	
Reperfusion therapeutic								
Glucose‐to‐HbA1c ratio groups	Q1	17 (0.87)	–	–	<0.0001	–	–	0.2319
	Q2	25 (1.14)	1.313 (0.707–2.439)	0.3884		1.406 (0.513–3.851)	0.5074	
	Q3	48 (2.13)	2.473 (1.418–4.314)	0.0014		2.911 (1.119–7.572)	0.0285	
	Q4	98 (3.52)	4.152 (2.473–6.971)	<0.0001		2.235 (0.683–7.314)	0.1838	
Without a history of previous infarction								
Glucose‐to‐HbA1c ratio groups	Q1	52 (0.20)	–	–	<0.0001	–	–	0.3870
	Q2	77 (0.30)	1.461 (1.027–2.078)	0.0348		1.111 (0.626–1.973)	0.7188	
	Q3	104 (0.40)	1.956 (1.401–2.729)	<0.0001		1.295 (0.727–2.307)	0.3800	
	Q4	214 (0.84)	4.128 (3.047–5.592)	<0.0001		1.369 (0.662–2.831)	0.3968	

^a^ Adjusted for age, sex, BMI, NIHSS on admission, hypertension, atrial fibrillation, antiplatelet, anticoagulation, lipid‐lowering drug, smoking, alcohol, LDL‐C, FBG, HbA1c, eGFR, HCY, systolic blood pressure, and diastolic blood pressure.

^b^ Test for trend based on the variable containing the median value for each quintile.

### 3.4 ROC analysis of SHR for predicting in‐hospital death

**FIGURE 2:**
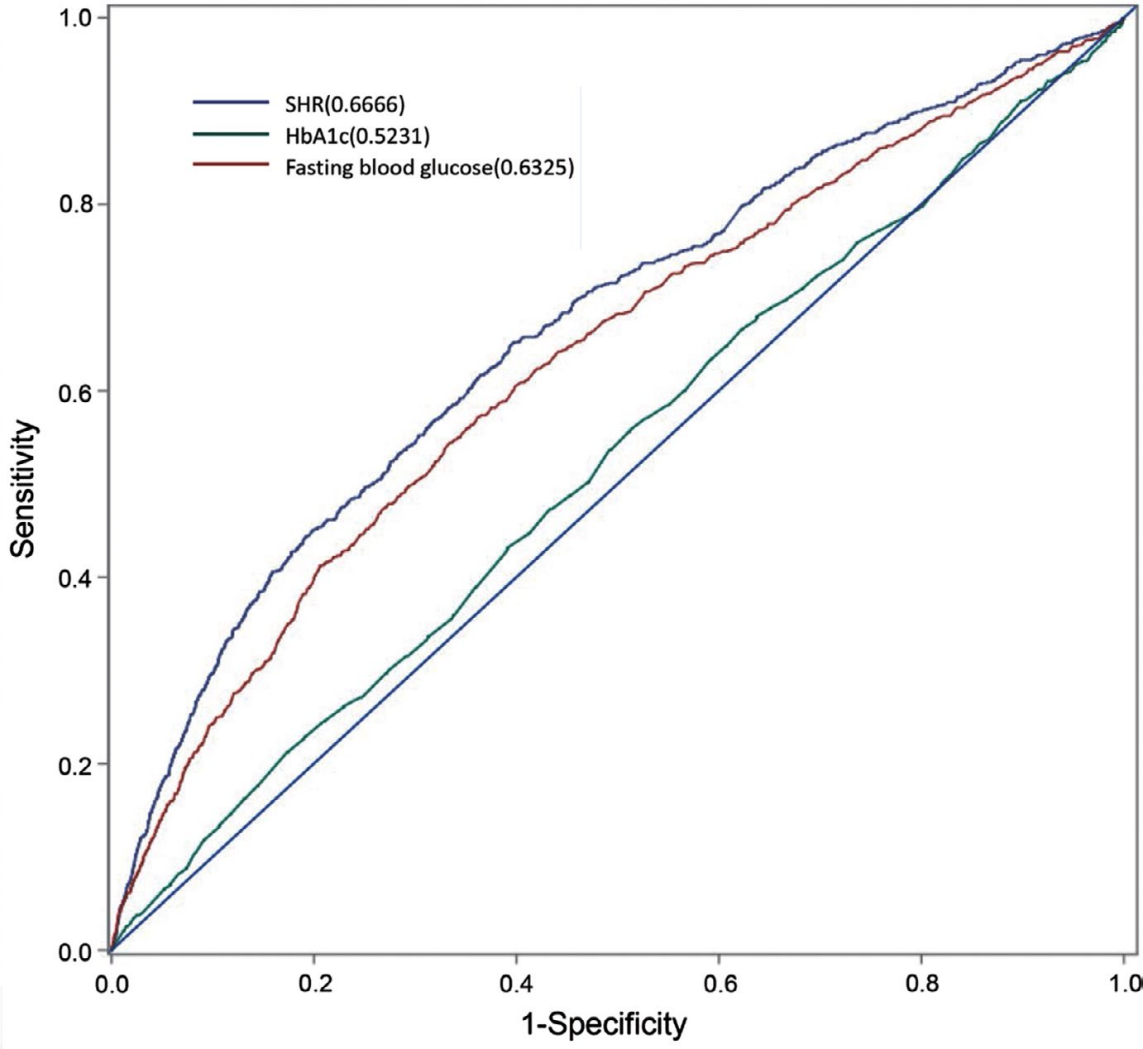
Receiver operating characteristic (ROC) analysis of stress‐induced hyperglycemia ratio (SHR), fasting blood glucose, and HbA1c for predicting in‐hospital death

The ROC analyses showed that SHR (AUC = 0.667, 95% CI: 0.647–0.686; 65.1% sensitivity, and 60.4% specificity) had a better predictive value for mortality than fasting blood glucose (AUC = 0.633, 95% CI: 0.613–0.652; 54.3% sensitivity, and 66.7% specificity) and HbA1c (AUC = 0.523, 95% CI: 0.504–0.543; 55.8% sensitivity, and 48.8% specificity) (Figure 2).

## 4 DISCUSSION

Stress hyperglycemia can occur in diabetic patients with acute severe cerebrovascular disease,^1^, ^3^, ^11^, ^14^ but the results regarding its association with stroke outcomes are conflicting. Therefore, this study aimed to examine the association between stress‐induced hyperglycemia and the occurrence of in‐hospital death in patients with diabetes and acute ischemic stroke. The study used the data from the CSCA, which is a national initiative to improve the burden of stroke in China.^21^ The results suggest that the SHR may serve as an accessory parameter for the prognosis of patients with diabetes after acute ischemic stroke.

Previous studies showed that stress hyperglycemia is associated with poor outcomes in patients with severe illnesses such as acute myocardiac infarction, trauma, and acute ischemic stroke.^6^, ^8^, ^9^ Zarean et al.^23^ showed that the glycemic gap was associated with mortality in patients with diabetes and hemorrhagic stroke. Nevertheless, the association between stress hyperglycemia and acute ischemic stroke in patients with diabetes was rarely reported. Several studies showed that admission hyperglycemia was significantly associated with poor short‐term outcomes of ischemic stroke after mechanic thrombectomy.^15^, ^16^, ^17^ Nevertheless, these studies did not further analyze the association of admission hyperglycemia with the outcome of acute ischemic stroke in patients with diabetes. It is believed that acute hyperglycemia has a distinct association with the increased risk of poor outcome or mortality in non‐diabetic patients.^3^, ^24^, ^25^ For patients with pre‐diabetes history, this relationship is controversial. Because hyperglycemia is a common trait among patients with DM, absolute hyperglycemia cannot reflect the changes in glucose under critical conditions without consideration of the basic glucose level, especially when blood glucose is poorly controlled. This might be a reasonable explanation for the paradoxical relationship between absolute hyperglycemia and mortality in patients with diabetes.^3^, ^7^ Therefore, the relative stress hyperglycemia indicators of glycemic gap and SHR, which take background blood glucose into consideration, should have a better prediction of the poor outcome of critical illness. By using the glycemic gap, Zarean et al.^23^ could exclude the impact of poor glycemic control in the outcome of diabetic patients with hemorrhagic stroke. Lee et al.^26^ showed that SHR could predict the in‐hospital mortality in critically ill patients across the glycemic spectrum, while absolute glycemia could not. A recent study showed that the SHR was a predictor of poor outcomes in non‐diabetic patients, but that study included only 18.1% (29/160) of diabetic patients.^27^ Merlino et al.^28^ showed in 414 patients (irrespective of the diabetes status) that stress hyperglycemia was associated with poor outcomes in patients with acute ischemic stroke after intravenous thrombolysis and that the quartiles of SHR had significant trends for poor outcomes and mortality. In patients with minor stroke or TIA, patients with newly diagnosed diabetes had a risk of recurrence similar to patients with known diabetes, while patients with stress hyperglycemia had a markedly higher risk of recurrence.^29^ Yuan et al.^10^ reported that stress hyperglycemia was associated with the risk of hemorrhagic transformation, which could explain, at least in part, the higher mortality observed with high SHR. In the present study, the glucose‐to‐HbA1c ratio was used, which also accounts for glycemic control. Indeed, blood glucose levels fluctuate widely during the course of the day and with disease conditions, while HbA1c levels represent the general glycemic control over the past 3 months.^13^ In addition, the AUC of SHR for mortality was higher than for FBG and HbA1c.

Although the underlying mechanisms are too complex to be fully understood, it was proposed that the hyperactivated oxidative stress response, insulin resistance, inflammation, cytokine production, and hormonal derangements may account for the association between stress hyperglycemia and poor outcomes.^11^, ^30^ The excess mortality has been hypothesized to be due to the combined effects of glucagon, growth factors, catecholamines, and glucocorticoids, leading to gluconeogenesis, inflammation, and insulin resistance,^31^ contributing to neuroinflammation and oxidative stress that exacerbates the brain injury.^32^ In addition, high blood glucose is associated with brain‐blood barrier breakdown, brain edema, and increased apoptosis.^33^, ^34^, ^35^ Furthermore, elevated blood glucose could aggravate inflammation and oxidative stress response, potentially creating a vicious cycle that leads to further hyperglycemia.^36^, ^37^ Meanwhile, hyperglycemia promotes the release of excessive circulating free fatty acids, which also aggravates hyperglycemia. The overlapping interaction of glucotoxicity, lipotoxicity, and inflammation might contribute to a detrimental physiopathological vicious cycle. In addition, hyperglycemia accelerates neuronal damage in hypoxic brain tissue.^38^ Hyperglycemia also increases the production of thrombin‐antithrombin complexes and the tissue factor pathway to stimulate coagulation.^39^ The activation of protein kinase C and NADPH oxidase increases reactive oxygen species (ROS) levels and reduces nitric oxide synthase, thereby leading to decreased reperfusion and possibly neuron damage.^39^ Evidence from MRI studies showed that admission hyperglycemia was associated with expanded infarction core and reduced penumbra salvage.^40^, ^41^ Therefore, the multiple intricate molecular mechanisms and pathological changes might lead to poor outcomes after stroke with stress hyperglycemia. These mechanisms probably affect nerve repair after stroke, impair collateral circulation, increase vascular permeability, increase platelet cohesion, and increase the occurrence of complications in patients. The exact mechanisms will have to be examined.

Still, poor glycemic control has been shown to be associated with poor functional outcomes after stroke,^42^ indicating that long‐term glycemic stress and damage are involved in the functional prognosis of stroke, while acute hyperglycemia after stroke might be a predictor of death. Unfortunately, the present study had no data about the functional outcomes after stroke. This will have to be examined in future studies.

Even though stress hyperglycemia is associated with poor outcomes after stroke, intensive glycemic control is not actually recommended during the acute phase. So far, no evidence showed that tight blood glucose control was associated with better outcomes after acute ischemic stroke. A meta‐analysis including 1296 patients with acute ischemic stroke from seven trials demonstrated that tight blood glucose control (i.e., maintaining glucose levels between 4.0 and 7.5 mmol/L) increased the risk of hypoglycemia events compared with the control group (OR = 25.9, 9.2–72.7).^43^ The current stroke guidelines recommend that blood glucose be controlled between 140 and 180 mg/dl,^44^ but the guidelines do not make any distinction between patients with or without diabetes history. The recently completed phase 3 trial Stroke Hyperglycemia Insulin Network Effort (SHINE) demonstrated that intensive blood glucose control (80–130 mg/dl) did not improve the functional outcomes and even increased the risk of severe hypoglycemia.^45^ To be worth mentioning, about 80% of the included patients had a history of diabetes. In view of the risks for diabetic patients, SHR is more meaningful than absolute hyperglycemia. Such a view is supported by a study in critically ill patients.^26^ As suggested by a recent review, acute management of stroke could include aggressive glucose management in patients with ischemic stroke and hyperglycemia.^46^

The present study has several limitations. First, we did not assess the blood glucose control during hospitalization because such data are not included in the database. Second, the design of this study was observational, and a cause‐effect relationship cannot be determined. Although it is a national multi‐center registration study with a huge amount of data, most of the enrolled patients have mild stroke severity, which makes the study inevitable choice bias. Third, no distinction is made between types 1 and 2 diabetes in the database. Thus, a prospective randomized large‐scale study is expected to clarify this relationship.

## 5 CONCLUSION

This study showed that the SHR is significantly associated with an increased risk of in‐hospital mortality in diabetic patients after acute ischemic stroke. This finding suggests that careful glycemic management is important for diabetic patients after stroke onset.

## CONFLICT OF INTEREST

All authors declare that they have no competing interests.

## AUTHOR CONTRIBUTIONS

DHM and ZXL wrote the manuscript and interpreted the data. HQG and YYJ contributed to review the manuscript and statistical problems. XQZ and YLW reviewed the manuscript. YJW interpreted data, reviewed and edited the manuscript, and contributed to discussions. All authors read and approved the final manuscript.

## ETHICS APPROVAL AND CONSENT TO PARTICIPATE

Written informed consent was obtained from all participants prior to data collection. The study and consent form details were approved by the Ethics Committee of Beijing Tiantan Hospital. This study adhered to the World Medical Association's Declaration of Helsinki (1964–2008) for Ethical Human Research, including confidentiality, privacy, and data management.

## Data Availability

The analysis data are owned by China National Clinical Research Center for Neurological Diseases (http://paper.ncrcnd.ttctrc.com/). Data are not available at present but upon reasonable request and with permission.
